# Correction: Ring-opening yields and auto-oxidation rates of the resulting peroxy radicals from OH-oxidation of α-pinene and β-pinene

**DOI:** 10.1039/d3ea90045b

**Published:** 2023-11-27

**Authors:** Ben H. Lee, Siddharth Iyer, Theo Kurtén, Jonathan G. Varelas, Jingyi Luo, Regan J. Thomson, Joel A. Thornton

**Affiliations:** a Department of Atmospheric Sciences, University of Washington Seattle WA USA joelt@uw.edu; b Aerosol Physics Laboratory, Tampere University Tampere Finland; c Department of Chemistry, University of Helsinki Helsinki Finland; d Department of Chemistry, Northwestern University Evanston IL USA

## Abstract

Correction for ‘Ring-opening yields and auto-oxidation rates of the resulting peroxy radicals from OH-oxidation of α-pinene and β-pinene’ by Ben H. Lee *et al.*, *Environ. Sci.: Atmos.*, 2023, **3**, 399–407, https://doi.org/10.1039/D2EA00133K.

The authors regret that a grant was missing in the acknowledgements section of the published article. The corrected acknowledgements section is shown below:

**Acknowledgements**

This work was funded by National Science Foundation Environmental Chemical Sciences (grant no. CHE-1807204 and CHE-2003359) and the European Research Council under the European Union's Horizon 2020 research and innovation programme under Grant No. 101002728. The authors acknowledge Ezra Wood (Drexel University) for his instructions on HO_*x*_ generation, and Henrik G. Kjærgaard (University of Copenhagen) and Kristian H. Møller (University of Copenhagen) for their suggestions on theoretical calculations.

The Royal Society of Chemistry apologises for these errors and any consequent inconvenience to authors and readers.

## Supplementary Material

